# Agroforestry leads to shifts within the gammaproteobacterial microbiome of banana plants cultivated in Central America

**DOI:** 10.3389/fmicb.2015.00091

**Published:** 2015-02-11

**Authors:** Martina Köberl, Miguel Dita, Alfonso Martinuz, Charles Staver, Gabriele Berg

**Affiliations:** ^1^Institute of Environmental Biotechnology, Graz University of TechnologyGraz, Austria; ^2^Brazilian Agricultural Research Corporation – EmbrapaBrasília, Brazil; ^3^Bioversity International Costa RicaTurrialba, Costa Rica; ^4^Bioversity International FranceMontpellier, France

**Keywords:** agroforestry, banana-associated *Gammaproteobacteria*, banana-coffee intercropping, Gros Michel, *Musa*

## Abstract

Bananas (*Musa* spp.) belong to the most important global food commodities, and their cultivation represents the world's largest monoculture. Although the plant-associated microbiome has substantial influence on plant growth and health, there is a lack of knowledge of the banana microbiome and its influencing factors. We studied the impact of (i) biogeography, and (ii) agroforestry on the banana-associated gammaproteobacterial microbiome analyzing plants grown in smallholder farms in Nicaragua and Costa Rica. Profiles of 16S rRNA genes revealed high abundances of *Pseudomonadales, Enterobacteriales, Xanthomonadales*, and *Legionellales*. An extraordinary high diversity of the gammaproteobacterial microbiota was observed within the endophytic microenvironments (endorhiza and pseudostem), which was similar in both countries. Enterobacteria were identified as dominant group of above-ground plant parts (pseudostem and leaves). Neither biogeography nor agroforestry showed a statistically significant impact on the gammaproteobacterial banana microbiome in general. However, indicator species for each microenvironment and country, as well as for plants grown in *Coffea* intercropping systems with and without agri-silvicultural production of different *Fabaceae* trees (*Inga* spp. in Nicaragua and *Erythrina poeppigiana* in Costa Rica) could be identified. For example, banana plants grown in agroforestry systems were characterized by an increase of potential plant-beneficial bacteria, like *Pseudomonas* and *Stenotrophomonas*, and on the other side by a decrease of *Erwinia*. Hence, this study could show that as a result of legume-based agroforestry the indigenous banana-associated gammaproteobacterial community noticeably shifted.

## Introduction

*Musa* spp., including dessert and cooking bananas, are large perennial monocotyledonous herbs of the order *Zingiberales*. Their domestication process started about 7000 years ago and involved hybridizations between diverse species and subspecies and the selection of sometimes diploid, but generally triploid seedless, parthenocarpic hybrids, which were thereafter widely dispersed by vegetative propagation (Perrier et al., 2011). The cultigens are landraces and belong to the most important agricultural crops in the tropics and sub-tropics. Worldwide, over 100 million metric tons of fruits are produced annually. Cultivars that enter international commerce are worth $5 billion per year, and locally consumed fruits are major staples for 400 million people in Latin America and Africa (FAOSTAT, 2005).

The *Musa acuminata* cultivar Gros Michel, also known as Big Mike, was the main exported banana variety from the nineteenth century until the late 1950s. However, in response to the susceptibility of this cultivar to the fungal pathogen *Fusarium oxysporum* f. sp. *cubense* (Foc) race 1, Gros Michel was widely replaced by the resistant Cavendish variety (Ploetz, 2006; Butler, 2013). In many countries in Central America, such as Costa Rica and Nicaragua, the Gros Michel variety is still grown, mainly by smallholder farmers in banana-coffee intercropping systems, sometimes in combination with agroforestry systems, where a lower disease incidence is reported in comparison to monocultures. Gros Michel fruits are praised for their fabulous flavor and, due to their thicker skin, for a better robustness to bruises in comparison to Cavendish. Agroforestry in general is a collective name for land-use systems in which woody perennials are grown in association with herbaceous plants or livestock, in spatial arrangement, a rotation or both (Lundgren, 1982). These practices are considered as functionally biodiverse, environmentally friendly and sustainable land-use alternatives. It was shown that such systems were able to enhance soil fertility and productivity by improving certain soil physical properties and protective functions, such as nutrient cycling and carbon sequestration (Montagnini and Nair, 2004; Seobi et al., 2005; Udawatta et al., 2009). Undeniably, it can be assumed that these environmental benefits are associated with soil microbial activity and soil biological parameters. In addition, the plant-associated microbiome has substantial influence on plant growth, quality, and health (Berg et al., 2014). However, despite the importance of banana for grower's livelihoods on these agroforestry systems in Central America and the hypothesized role of soil and plant microbiome on building healthier environments, knowledge on the microbial diversity of representative productions areas is still scarce.

The objective of this study was to decipher the gammaproteobacterial microbiome of banana plants cultivated in Central America. In order to obtain an almost complete picture of the banana-colonizing *Gammaproteobacteria* under diverse conditions, different plant parts and microenvironments were investigated: the rhizosphere soil surrounding the roots and represents the interface to the bulk soil, the inner tissue of the roots - the endorhiza, the banana leaves, as well as the pseudostem. The cylindrical succulent pseudostem is a peculiarity of the herbaceous banana plant which consists of closely packed leaf-petiole sheaths (Saravanan and Aradhya, 2011). It provides a unique microhabitat for endophytic microorganisms and was recently identified as a bacterial hot spot colonized by an extraordinary high abundance and diversity of enterics (Rossmann et al., 2012). Consequently, we hypothesized a key role of the *Enterobacteriaceae* for plant health especially in the endophytic microenvironments. To additionally capture the group of often plant-beneficial fluorescent pseudomonads (Ayyadurai et al., 2006; Weller, 2007), and at the same time to preserve the necessary sequencing depth, we decided to focus on the whole gammaproteobacterial fraction by employing a comprehensive 16S rRNA gene amplicon sequencing approach. Comparisons between colonization patterns reveal the impact of (i) biogeography (Nicaragua vs. Costa Rica), and (ii) agroforestry conditions (banana-coffee intercropping with vs. without agroforestry) on the banana-associated gammaproteobacterial microbiota.

## Materials and methods

### Experimental design and sampling procedure

Samples were taken in November 2012 from *M. acuminata* Colla (AAA group) cultivar Gros Michel in Nicaragua and Costa Rica. In each country, samples of banana roots, pseudostem, leaves, and rhizosphere soil were collected from three different farms (Figure S1), where bananas were cultivated in intercropping systems with *Coffea* spp. To understand the effect of agroforestry on the banana-associated microbiome, samples were collected on each farm from sites with and without associated *Fabaceae* trees. The predominant trees were *Inga* spp. in Nicaragua and *Erythrina poeppigiana* in Costa Rica. Each site was under the respective production system since more than 50 years. Composite samples consisting of sub-samples from five appropriate plants without visible infestation of any disease were taken for each microenvironment.

### Total community DNA isolation

For extraction of metagenomic DNA from the rhizosphere, 2 g of rhizospheric soil were mixed with 15 ml of 0.85% NaCl for 10 s on the vortex. To isolate total community DNA from the endorhiza, 5 g of roots were surface-sterilized with 4% NaOCl for 5 min. Afterwards, roots were washed three times with sterile distilled water and transferred to sterile WhirlPaks (Nasco, Fort Atkinson, USA), then 10 ml of 0.85% NaCl were added and the surface-sterilized roots were homogenized using mortar and pestle. Pseudostem samples (5 g) were washed with sterile distilled water, transferred to WhirlPaks, and after 10 ml of 0.85% NaCl were added, homogenized with mortar and pestle. From phyllosphere samples, 5 g of leaves were washed three times with sterile distilled water, before homogenization with 10 ml of 0.85% NaCl. From the liquid parts 4 ml were centrifuged at high speed (16,000 × g, 4°C) for 20 min and resulting pellets were stored at −70°C. Total community DNA was extracted using the FastDNA SPIN Kit for Soil (MP Biomedicals, Solon, USA) according to the manufacturer's protocol. Metagenomic DNA samples were encoded using abbreviations indicating: (1) country (N−, Nicaragua; C−, Costa Rica), (2) microenvironment (S, rhizosphere soil; Re, endorhiza; Ps, pseudostem; L, leaves), (3) farm (1–3 in each country; Figure S1) (4) agroforestry conditions (T+, with trees; T−, without trees).

### Gammaproteobacterial 16S rRNA gene profiling by Illumina MiSeq sequencing

For a deep-sequencing analysis of the banana-associated *Gammaproteobacteria* community, the hypervariable V4 region of the 16S rRNA gene was amplified in a nested PCR approach with the *Gammaproteobacteria* specific primer pair Gamma395f/Gamma871r (Mühling et al., 2008) and the universal primer pair 515F/806R (Caporaso et al., 2011), which carried sample specific tags. The reaction mixture for the first PCR (20 μl) contained 1 × Taq&Go (MP Biomedicals, Eschwege, Germany), 2 mM MgCl_2_, 0.1 μM of each primer and 1 μl of template DNA dilution (96°C, 4 min; 30 cycles of 96°C, 1 min; 54°C, 1 min; 74°C, 1 min; and elongation at 74°C, 10 min). The second PCR (30 μl) was performed by using 1 × Taq&Go, 0.2 μM of each primer and 1.2 μl from dilutions of the first PCR mixtures (94°C, 3 min; 32 cycles of 94°C, 45 s; 60°C, 1 min; 72°C, 18 s; and elongation at 72°C, 10 min). PCR products of three independent reactions were pooled in equal volumes and purified by employing the Wizard SV Gel and PCR Clean-Up System (Promega, Madison, USA). Sequence libraries were generated by a paired-end approach using the Illumina MiSeq platform (Eurofins MWG, Ebersberg, Germany). The nucleotide sequences are available in the European Nucleotide Archive (www.ebi.ac.uk/ena) under the accession number PRJEB8107.

Data analysis was performed by employing the open source software package QIIME 1.8 (Caporaso et al., 2010a). Sequencing reads with more than three consecutive low quality base calls (Phred quality score ≤ 20) were truncated at the position where their quality began to drop, and only reads with >75% consecutive high quality base calls, without any ambiguous characters, and longer than 200 nucleotides in length were retained for further analyses. All quality sequences were adjusted in the same orientation and clustered into operational taxonomic units (OTUs) with uclust (Edgar, 2010), using 3, 5, and 10% dissimilarity thresholds. From each OTU the most abundant sequence was selected as the representative one, and the taxonomy of the representative set was assigned with the uclust-based consensus taxonomy assigner using an 80% confidence threshold. The representative sequence set was aligned with PyNAST (Caporaso et al., 2010b). Chimera check was performed with ChimeraSlayer and potentially chimeric sequences were discarded. OTU tables at the different dissimilarity levels were constructed, and OTUs not assigned to the class of *Gammaproteobacteria* as well as singletons were removed from the dataset. For alpha and beta diversity analyses, OTU tables were rarefied at 13,610 reads. Diversity indices Shannon (Shannon, 1997) and Chao1 (Chao and Bunge, 2002) were determined based on the normalized clustering data. Significant differences were calculated with PASW Statistics 18 (SPSS Inc., Chicago, IL, USA) using Tukey and Games-Howell *post hoc* tests, depending on the homogeneity of variances. Beta diversity was analyzed based on weighted UniFrac distances (Lozupone et al., 2007) and 10 jackknife replicates of the total rarefied datasets. Statistical analyses were performed using the adonis test with 999 permutations. Taxonomy based ring-charts were created with Krona 2.2 (Ondov et al., 2011).

Profile clustering network analyses were performed in order to highlight single taxonomic groups corresponding to genus level (OTUs at a dissimilarity level of 3% summarized at taxonomic level 6) with considerable differences between banana plants grown in Nicaragua and in Costa Rica and between those grown with and without associated trees. The network analyses were carried out with taxa exhibiting a mean read change of more than 0.2% of the data set. If the ratio of relative mean abundances exceeded 1.5, the taxa were regarded as altered and assigned to the respective profile. Networks depicting community changes resulting from biogeographical location were restricted to taxa which significantly differed between countries. Significant differences were ascertained with Metastats (White et al., 2009), where *p*-values were computed using a combination of the non-parametric *t*-test, exact Fisher's test, and the false discovery rate with 10^3^ permutations. For networks showing differences caused by agroforestry, only taxonomic groups featuring the same pattern in all three farms of a country were considered. Visualization of the networks was carried out using Cytoscape 2.8.3 (Smoot et al., 2011).

## Results

### Richness and diversity of the banana-associated gammaproteobacterial community

**Figure 1:**
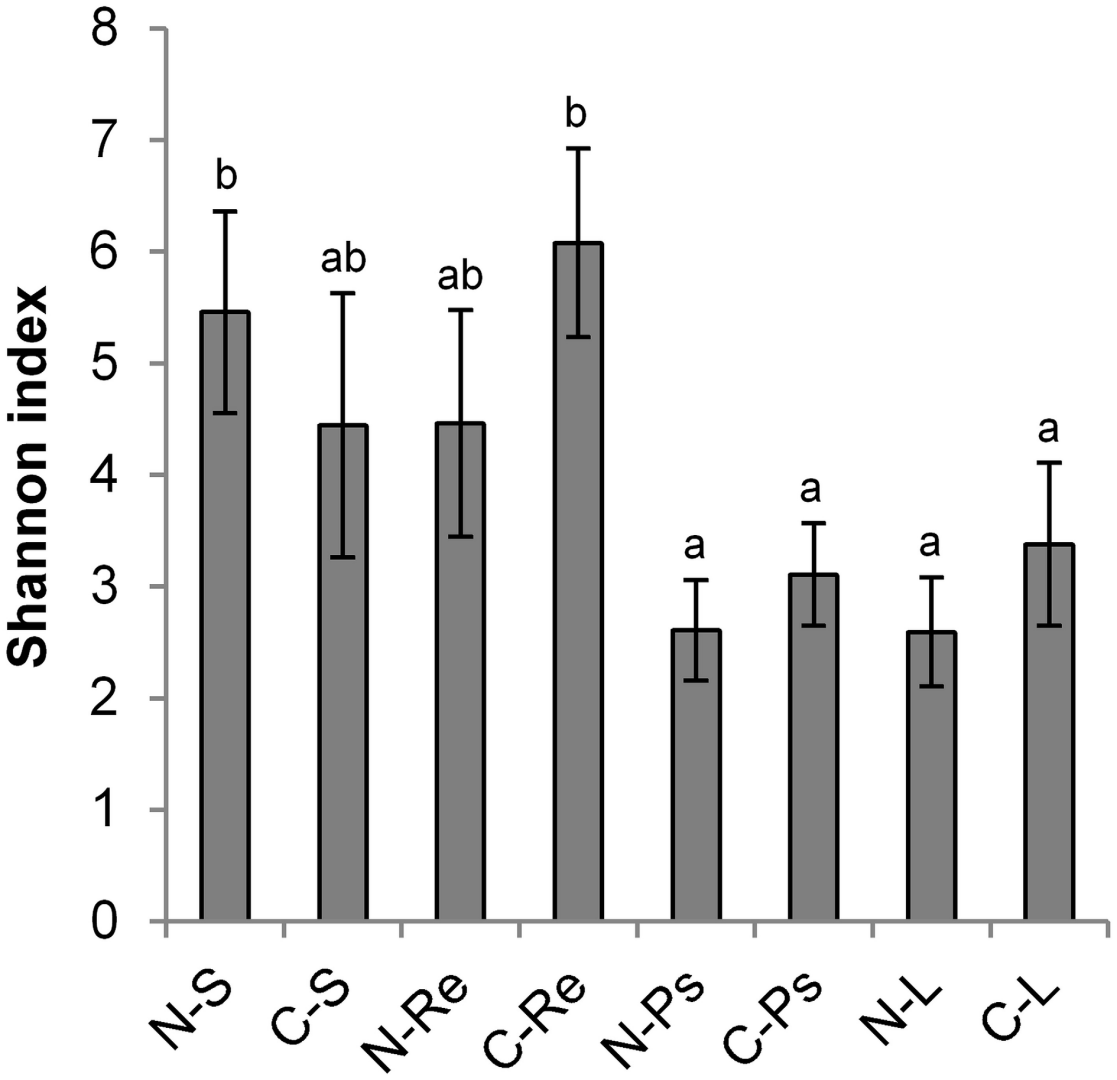
**Shannon indices of gammaproteobacterial diversity for different microenvironments (S, rhizosphere soil; Re, endorhiza; Ps, pseudostem; L, leaves) at a genetic distance of 3%, separated in samples from Nicaragua (N−) and Costa Rica (C−)**. Data were ascertained by 16S rRNA gene profiling and are averages of the three sampling farms of a country ± confidence. Significant differences (*p* ≤ 0.05, Tukey *post hoc* test) are indicated by lowercase letters.

The gammaproteobacterial microbiota associated to the rhizosphere, endorhiza, pseudostem, and foliage of healthy banana plants grown under different agroforestry conditions in Nicaragua and Costa Rica analyzed by a barcoded 16S rRNA gene amplicon sequencing approach based on Illumina MiSeq sequencing yielded in 2,234,043 quality sequences with a read length ≥200 nucleotides, between 13,619 and 111,332 quality reads per sample. Rarefaction analyses of the sequencing libraries at a genetic dissimilarity level of 3% are depicted in Figure S2. Comparisons of observed OTUs with their estimated richness by the Chao1 index revealed coverage between 87.3 and 47.4% per sample at order level (Table S1). The sequencing efforts at genus and species level reached 74.1–39.9% and 68.8–31.5%, respectively. The computed Shannon indices of diversity (H′) ranged from 7.56 to 1.47 at a genetic distance of 3% (Table S1). In general, rhizosphere and endorhiza samples exhibited higher gammaproteobacterial diversity than pseudostem and leaves (Figure 1). Within samples from Nicaragua, the highest values were observed for the rhizospheric soil (5.46 on average ± 0.90 confidence), but without a significant difference (*p* ≤ 0.05, Tukey *post hoc* test) to the endorhiza (4.46 ± 1.02). Significantly lower Shannon indices than in the rhizosphere soil were detected for pseudostem (2.61 ± 0.45) and leaves samples (2.59 ± 0.49). Banana plants from Costa Rica revealed the highest diversity in the endorhiza (6.08 ± 0.85), which not significantly differed from the rhizosphere soil (4.45 ± 1.18). Significantly lower values than in the endorhiza were observed for leaves (3.38 ± 0.73) and pseudostem (3.11 ± 0.46). Between the same microenvironments of banana plants from the two countries, no significant differences were observed. Agroforestry did not show a significant impact (*p* ≤ 0.05, Games-Howell *post hoc* test) on the gammaproteobacterial diversity of the different microenvironments.

### Taxonomic composition of the gammaproteobacterial banana microbiome

**Figure 2:**
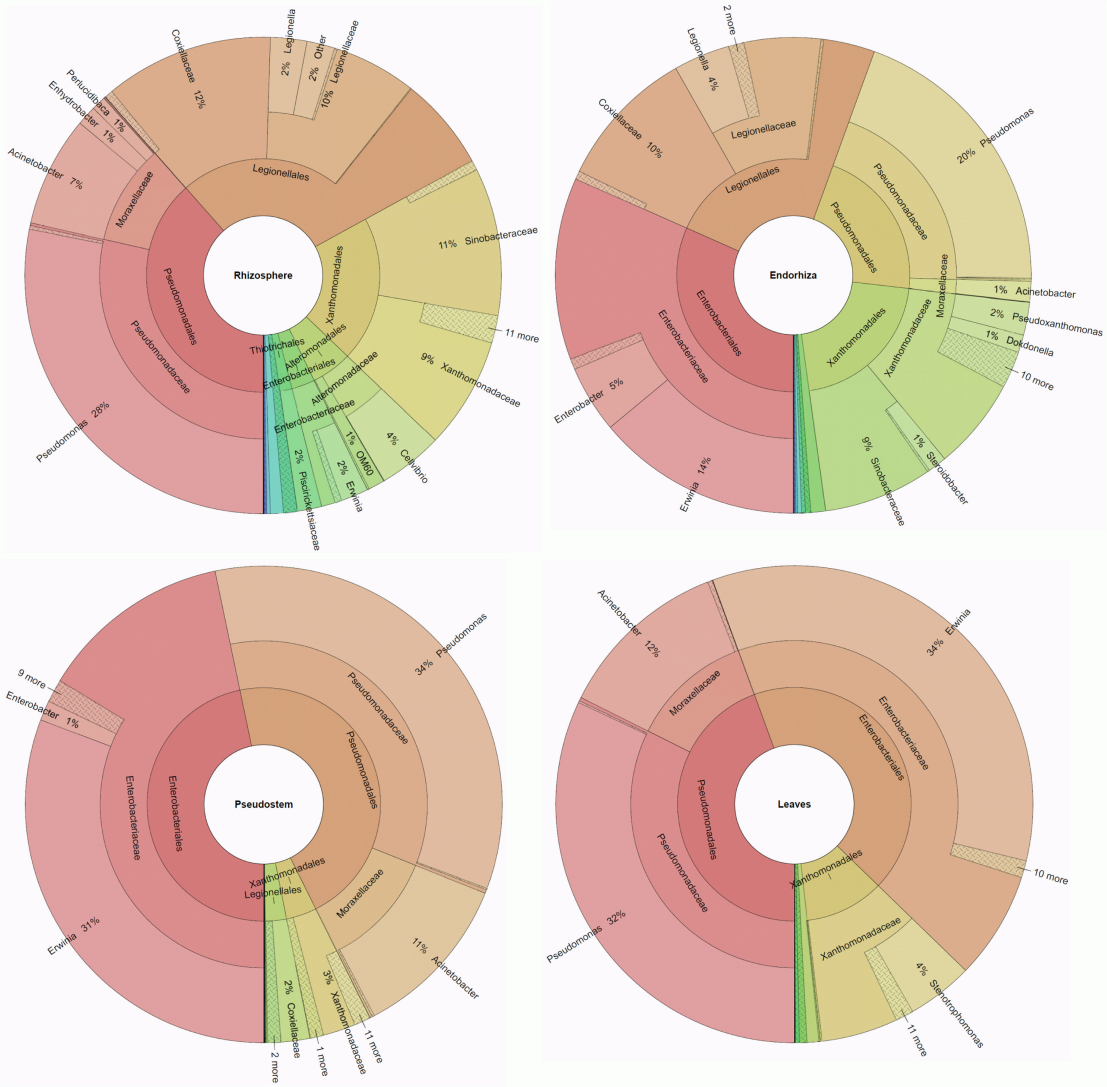
**Taxonomic composition of the gammaproteobacterial banana microbiome**. Ring-charts for each microenvironment depict the mean values of 12 composite samples from healthy plants grown on six different smallholder farms in Nicaragua and Costa Rica, independent of their agroforestry conditions and biogeographical differences.

**Figure 3:**
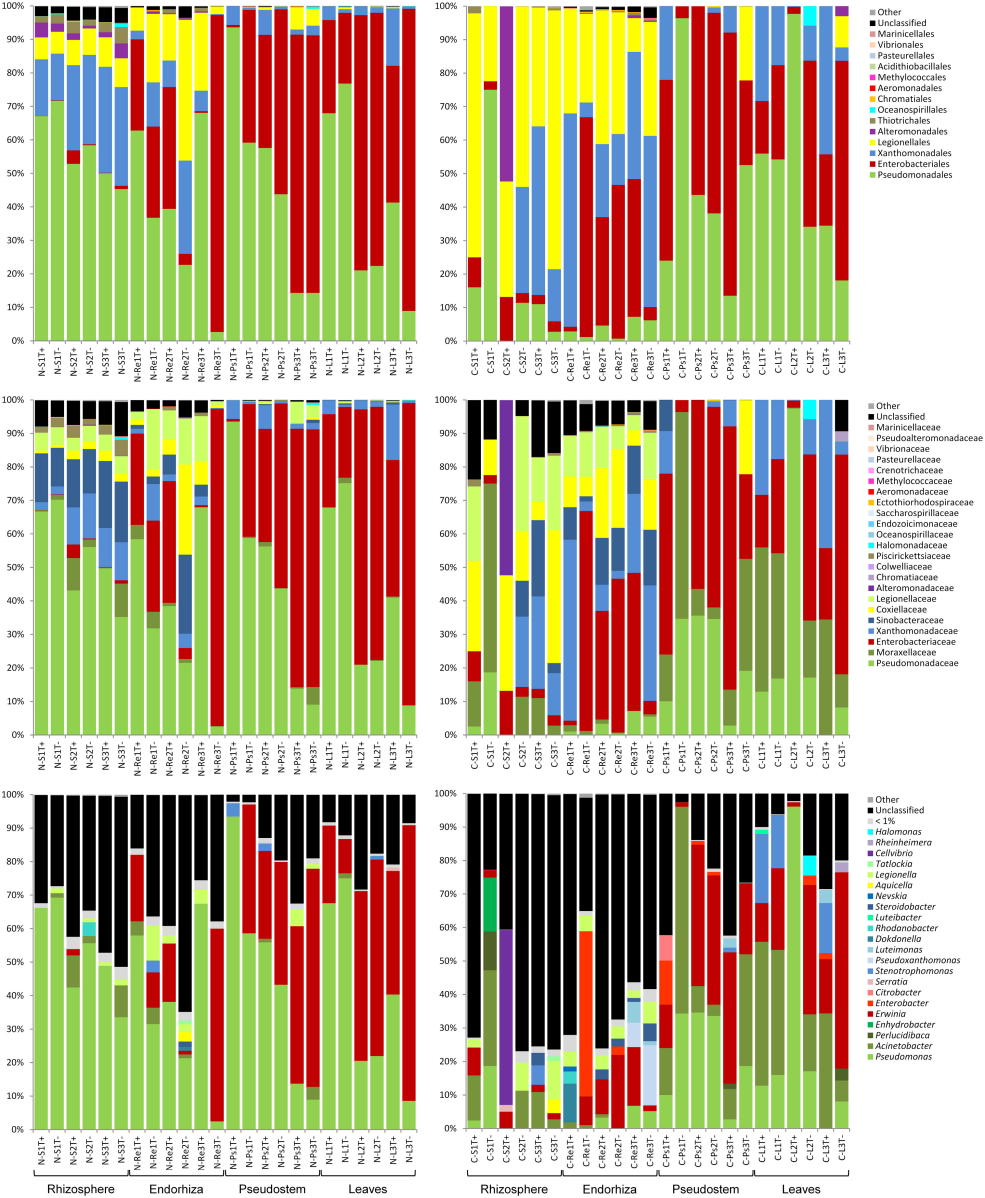
**Taxonomic composition of *Gammaproteobacteria* communities inhabiting rhizosphere, endorhiza, pseudostem, and leaves of banana plants from Nicaragua (left) and Costa Rica (right) grown under different agroforestry conditions**. Sequences obtained by Illumina MiSeq sequencing were classified at order, family and genus level. Samples were encoded using abbreviations indicating: (1) country (N−, Nicaragua; C−, Costa Rica), (2) microenvironment (S, rhizosphere soil; Re, endorhiza; Ps, pseudostem; L, leaves), (3) farm (1, 2, 3), and (4) agroforestry conditions (T+, with trees; T−, without trees).

Nearly all quality sequences could be assigned below the class level, and over all banana-associated communities, high abundances of *Pseudomonadales, Enterobacteriales, Xanthomonadales*, and *Legionellales* were found (Figures 2, 3). The rhizosphere of bananas from Nicaragua was colonized by a significantly higher abundance (*p* ≤ 0.05, Metastats) of *Pseudomonadales, Thiotrichales*, as well as of unclassified *Gammaproteobacteria* than the rhizosphere soil of Costa Rica. Conversely, the plant rhizosphere from Costa Rica was inhabited to a greater extent of *Legionellales* and *Enterobacteriales*. The endorhiza of bananas from Nicaragua exhibited significantly higher relative abundances of *Pseudomonadales*, while *Xanthomonadales* occurred in higher abundances in endorhiza samples from Costa Rica. The pseudostem in general was highly dominated by *Enterobacteriales* and *Pseudomonadales* and showed no significant differences between countries at order level. The foliage exhibited a similar gammaproteobacterial colonization to the pseudostem. However, the leaves from Costa Rica revealed in addition to the dominant orders higher abundances of *Oceanospirillales* than those from Nicaragua.

At lower taxonomic levels, *Pseudomonadales* could be assigned to *Pseudomonadaceae* (genus *Pseudomonas*) and *Moraxellaceae* (genera *Acinetobacter, Perlucidibaca*, and *Enhydrobacter*), whereby in general Nicaragua samples were highly dominated by *Pseudomonadaceae* and samples from Costa Rica revealed a high abundance of *Moraxellaceae*. The enterobacterial fraction was dominated by *Erwinia* with lower abundances of *Enterobacter, Citrobacter*, and *Serratia*. *Xanthomonadales* sequences could be assigned to different *Xanthomonadaceae* (*Stenotrophomonas, Pseudoxanthomonas, Luteimonas, Dokdonella, Rhodanobacter*, and *Luteibacter*) and *Sinobacteraceae* (*Steroidobacter*, and *Nevskia*). *Legionellales* could be divided into the families *Coxiellaceae* (*Aquicella*) and *Legionellaceae* (*Legionella*, and *Tatlockia*). Further genera identified for taxonomic groups with a relative abundance over 1% per sample belonged to *Alteromonadales* (*Cellvibrio*, and *Rheinheimera*) and to *Oceanospirillales* (*Halomonas*).

### Impact of biogeography and agroforestry

**Figure 4:**
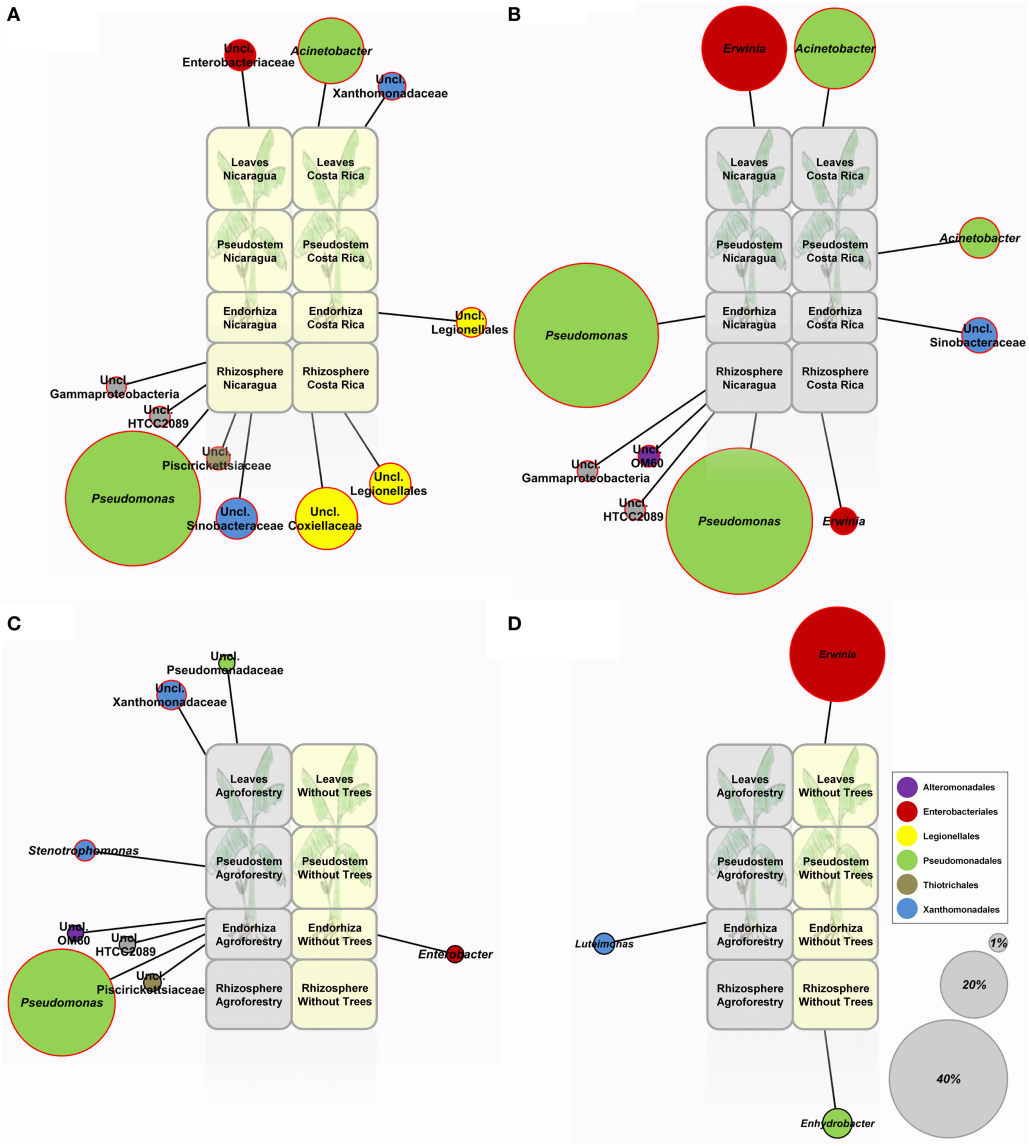
**Profile clustering network analyses depicting the impact of biogeography and agroforestry on the gammaproteobacterial microbiome of banana plants cultivated in Nicaragua and Costa Rica**. **(A)** Impact of biogeography without agroforestry, **(B)** Impact of biogeography and different agroforestry systems (*Inga* spp. in Nicaragua; *Erythrina poeppigiana* in Costa Rica), **(C)** Impact of agroforestry in Nicaragua, **(D)** Impact of agroforestry in Costa Rica. The relative abundances of OTUs at a dissimilarity level of 3% summarized at genus level with a mean read change between different conditions of more than 0.2% of the data set were used. If the ratio of relative mean abundances exceeded 1.5, the taxa were regarded as altered and assigned to the respective profile. Node sizes correspond to the abundance change between conditions; nodes matching to changes of 1, 20, and 40% of the data set were added as reference points. **(A,B)** Number of depicted nodes was reduced to taxa with significant differences between countries (*p* ≤ 0.05, Metastats). **(C,D)** Only those taxonomic groups that featured the same pattern in all three farms of the respective country are shown. Significant differences are indicated by red node borders.

Considering the total gammaproteobacterial community, no significant differences (*p* ≤ 0.05, adonis test) based on weighted UniFrac distances could be calculated for individual microenvironments between banana plants grown in Nicaragua and Costa Rica (Table S2), and for none of the countries a significant impact on the banana-colonizing *Gammaproteobacteria* resulting from tree presence was found (Table S3). However, profile clustering network analyses revealed differences of individual taxonomic groups in the colonization patterns between banana plants of the two Central American countries as well as between plants grown in agroforestry systems and those grown without associated trees (Figure 4). Each network subdivides the four investigated microenvironments (rhizosphere soil, endorhiza, pseudostem, and leaves), leaving out taxonomic groups without considerable differences between different conditions. In the networks visualizing the impact of biogeography, only taxa with significant differences (*p* ≤ 0.05, Metastats) between the sampling countries were shown, while in the networks depicting the impact of agroforestry, only taxa featuring the same pattern in all three farms of the respective country were considered.

Without the influence of different agroforestry trees, banana plants from Nicaragua revealed a significantly higher abundance of *Pseudomonas*, unclassified *Sinobacteraceae, Piscirickettsiaceae*, and other unclassified *Gammaproteobacteria* in their rhizosphere (Figure 4A), while the rhizosphere and also the endorhiza from plants in Costa Rica was colonized to a greater extent by *Legionellales* (unclassified *Coxiellaceae* and others). The pseudostem did not show significant differences in its gammaproteobacterial colonization between the two countries in plants grown without associated trees. However, the leaves from plants grown in Nicaragua exhibited higher numbers of unclassified *Enterobacteriaceae*, while those of plants from Costa Rica had higher abundances of *Acinetobacter* and unclassified *Xanthomonadaceae*. Under agroforestry conditions, the below-ground habitats of banana plants grown in Nicaragua in association with *Inga* spp. were characterized by much higher abundances of *Pseudomonas* than bananas cultivated under agroforestry conditions with *E. poeppigiana* in Costa Rica (Figure 4B). The rhizosphere of plants grown in the *Inga* agroforestry system further revealed higher abundances of unclassified *Alteromonadales* and other unclassified *Gammaproteobacteria*, while banana plants grown in the *Erythrina* agroforestry system were more inhabited by unclassified *Sinobacteraceae* in their endorhiza and by *Erwinia* in their rhizosphere. Conversely, banana leaves from the *Inga* agroforestry system in Nicaragua showed a significantly higher number of *Erwinia*, while the aerial plant parts of Costa Rica's bananas from the *Erythrina* agroforestry system were colonized to a greater extent by *Acinetobacter*. In comparison to banana plants grown without associated trees, plants cultivated in agro-ecosystems in Nicaragua harbored an increased number of *Pseudomonas* (species unclassified) in their endorhiza (Figure 4C), as well as of *Xanthomonadaceae* (*Stenotrophomonas* and others) in their above-ground parts. Costa Rica's plants grown in a system without trees revealed a significantly higher number of *Erwinia* in their phyllosphere than appropriate plants grown in an agroforestry system (Figure 4D).

## Discussion

A deep sequencing analysis of the gammaproteobacterial microbiome associated with the Gros Michel banana variety in Central America revealed an extraordinary high diversity within the endophytic community. Considering the below-ground microhabitats, the endorhiza of plants grown in Nicaragua unveiled a diversity comparable to that of the rhizosphere soil. The succulent pseudostem which can be considered as an above-ground endophytic microhabitat revealed a diversity comparable to that of the leaves encompassing endo- as well as ectophytes. A 16S rRNA gene amplicon sequencing approach targeting only the enterobacterial community of the banana plant revealed a strikingly diverse colonization of its endosphere with a Shannon diversity index for the pseudostem (H′ = 0.55) similar to those of rhizosphere samples (H′ = 0.40–0.55), even though based on only one pseudostem sample (Rossmann et al., 2012). But normally, what we know from other plants, we face a contrasting picture; due to root exudates and the resulting high nutrient content, the rhizosphere represents a favored microenvironment for microbial colonization and is characterized by a high abundance and diversity (Berendsen et al., 2012; Berg et al., 2014), and only a fraction of this root-associated microorganisms is able to invade, compete with other well-adapted endophytes, and successfully colonize the inner plant tissue (Germaine et al., 2004; Chi et al., 2005). Several endophytes are known for their advantageous associations and close interactions with their host plants. They have been shown to enhance plant growth and quality (Berg et al., 2005a; Köberl et al., 2013), increase plant resistance to abiotic stresses, pathogens and even herbivores (Rodriguez et al., 2009; Marasco et al., 2012; Yi et al., 2013), and contribute to plant-assisted bioremediation (Lodewyckx et al., 2001; Siciliano et al., 2001). The generally high diversity within the endophytic community of the banana plant can be explained by the permanent nature of its corm serving as a reservoir for endophytic diversity and the transmission to following generations via vegetative suckers.

In addition to the diversity, the gammaproteobacterial taxonomic composition was highly similar between the endophytic pseudostem and banana leaves as well, revealing a predominant colonization by *Enterobacteriaceae* and *Pseudomonadales*. *Pseudomonadales*, in particular the genus *Pseudomonas* but also *Acinetobacter* (both identified as dominant groups in this study), are well-known plant colonizers and among others often accountable for beneficial plant-microbe interactions (Weller, 2007; Rolli et al., 2015). Interestingly, while the *Pseudomonadales* community in samples from Nicaragua was highly dominated by the genus *Pseudomonas*, banana plants from Costa Rica revealed significantly higher relative abundances of *Acinetobacter*. Although it is well-known that the plant microbiome is shaped by both soil community and the plant cultivar (Berg and Smalla, 2009), the dominance of enterics in the banana pseudostem described for the East African Highland banana of Uganda (Rossmann et al., 2012) could be confirmed for the Gros Michel variety cultivated in Central America as well and could be extended to the entire perennial above-ground plant parts of the banana. However, while the colonization study of the East African Highland banana in Uganda (Rossmann et al., 2012) revealed *Enterobacter* as the predominant enterobacterial genus in plant-associated microenvironments, *Erwinia* was identified as the most dominant genus in the Central American Gros Michel variety. In contrast to *Enterobacter* which comprises several opportunistic human pathogenic strains (*E. aerogenes, E. cloacae*) (Berg et al., 2015), *Erwinia* is mainly known as plant pathogen (*E. amylovora, E. tracheiphila*) (Eastgate, 2000; Rojas et al., 2013). However, as this study encompassed only healthy banana plants without symptoms of *Fusarium* wilt or any other disease, there is no indication that the *Erwinia* strains observed within the banana-associated microbiome are in any manner harmful to the plant. A recent study of the lettuce (*Lactuca sativa*) microbiome also revealed a preferential occurrence of enterics in the phyllosphere (Erlacher et al., 2014).

In general, a higher impact on the banana-associated gammaproteobacterial microbiome was observed for the biogeographical location than for the agroforestry conditions. The biggest differences between the sampling countries were observed for the rhizosphere communities, representing the most probable source of all other plant colonizers. Consequently, based on the different rhizosphere microbiomes, disparities were found for all investigated microenvironments, whereby above-ground plant parts shared higher similarities, possibly due to a rigorous selection process with subsequent enrichment especially of enterics and pseudomonads. In addition to generally high contents of polyphenols and antioxidants in the succulent banana pseudostem, Saravanan and Aradhya (2011) could recently measure high concentrations of flavonoid compounds. Flavonoids are widely distributed secondary metabolites with diverse metabolic functions in plants; among several others, some of them are well-known for their antimicrobial activity (Falcone Ferreyra et al., 2012) and have been identified to be involved in the plant-driven selection of microbes (Bais et al., 2006; Weston and Mathesius, 2013).

For both countries and different agroforestry systems, a slight shift of the gammaproteobacterial microbiome resulting from associated *Fabaceae* trees could be observed. Banana plants grown in the agroforestry system with *Inga* trees in Nicaragua revealed significantly higher abundances of *Pseudomonas* and *Stenotrophomonas*. Both genera comprise several potential plant-beneficial species. For instance, *Stenotrophomonas rhizophila* has become a model bacterium among the plant growth-promoters and stress protecting agents (Alavi et al., 2013), particularly because of its beneficial effects on plants in salinated soils (Egamberdieva et al., 2011). Positive *Pseudomonas*-plant interactions are well-known (Weller, 2007) and have already been discussed. However, the genus *Pseudomonas* also includes some species with potential deleterious effects on plants (*P. syringae, P. viridiflava*) (Jakob et al., 2002), and moreover some species of *Pseudomonas* and also of *Stenotrophomonas* are known as opportunistic pathogens in humans as well (*P. aeruginosa, S. maltophilia*). Several studies provided evidence that similar or even identical functions are responsible for the beneficial interactions with plants and virulence in other eukaryotic hosts (Berg et al., 2005b; Alavi et al., 2014). For banana plants grown in association with *E. poeppigiana* in Costa Rica, a significant decrease of *Erwinia* spp. was recorded. Although this study targeted exclusively the gammaproteobacterial fraction, results could show that as a consequence of legume-based agroforestry the indigenous banana-associated microbial community was noticeably shifted.

## Author contributions

Conceived and designed the experiments: GB, CS, MD. Performed the experiments: MK, AM. Analyzed the data: MK, MD, GB. Contributed reagents/materials/analysis tools: CS, GB. Wrote the paper: MK, GB.

### Conflict of interest statement

The authors declare that the research was conducted in the absence of any commercial or financial relationships that could be construed as a potential conflict of interest.
