# Supplementary material for: Agroforestry leads to shifts within the gammaproteobacterial microbiome of banana plants cultivated in Central America
Source: Front Microbiol. 2015 Feb 11;6:91. doi: 10.3389/fmicb.2015.00091 (PMC4324142; doi:10.3389/fmicb.2015.00091)
Supplement: Supplementary file 1 [file DataSheet1.DOCX]

**Supplementary Material**

**Table S1** Richness estimates and diversity indices for gammaproteobacterial 16S rRNA gene amplicon libraries at 3%, 5% and 10% genetic dissimilarity.

| Sample^a^ | Clusters^c^ (OTUs) | | | Chao1 (OTUs) | | | Coverage (%) | | | Shannon (H’) | | |
| --- | --- | --- | --- | --- | --- | --- | --- | --- | --- | --- | --- | --- |
|  | 3% | 5% | 10% | 3% | 5% | 10% | 3% | 5% | 10% | 3% | 5% | 10% |
| N-S1T+ | 1,057 | 695 | 182 | 2,467 | 1,614 | 293 | 42.9 | 43.0 | 62.0 | 4.44 | 3.28 | 2.27 |
| N-S1T- | 1,294 | 814 | 199 | 3,648 | 1,786 | 293 | 35.5 | 45.6 | 68.0 | 4.15 | 3.39 | 2.30 |
| N-S2T+ | 1,586 | 1,044 | 267 | 4,223 | 2,422 | 391 | 37.5 | 43.1 | 68.2 | 5.69 | 4.77 | 3.54 |
| N-S2T- | 1,450 | 1,037 | 274 | 3,817 | 2,407 | 381 | 38.0 | 43.1 | 71.8 | 5.01 | 4.32 | 3.09 |
| N-S3T+ | 1,912 | 1,317 | 308 | 5,013 | 2,907 | 459 | 38.1 | 45.3 | 67.1 | 5.89 | 5.13 | 3.41 |
| N-S3T- | 2,455 | 1,693 | 331 | 5,731 | 3,383 | 445 | 42.8 | 50.0 | 74.4 | 7.56 | 6.59 | 4.30 |
| N-Re1T+ | 973 | 589 | 173 | 2,412 | 1,298 | 279 | 40.3 | 45.4 | 62.0 | 3.47 | 2.62 | 2.08 |
| N-Re1T- | 633 | 247 | 76 | 1,189 | 546 | 132 | 53.3 | 45.2 | 57.2 | 5.34 | 4.01 | 3.01 |
| N-Re2T+ | 912 | 596 | 171 | 1,541 | 965 | 223 | 59.2 | 61.7 | 76.5 | 4.84 | 3.66 | 2.66 |
| N-Re2T- | 713 | 515 | 164 | 1,399 | 881 | 251 | 51.0 | 58.5 | 65.6 | 5.93 | 5.28 | 4.02 |
| N-Re3T+ | 714 | 502 | 160 | 1,038 | 677 | 205 | 68.8 | 74.1 | 78.2 | 5.04 | 3.51 | 2.30 |
| N-Re3T- | 365 | 186 | 69 | 789 | 376 | 112 | 46.3 | 49.5 | 61.5 | 2.15 | 0.78 | 0.53 |
| N-Ps1T+ | 94 | 62 | 14 | 299 | 114 | 23 | 31.5 | 54.5 | 61.7 | 1.53 | 1.42 | 0.51 |
| N-Ps1T- | 276 | 111 | 39 | 581 | 205 | 64 | 47.5 | 54.3 | 60.8 | 2.23 | 1.32 | 1.14 |
| N-Ps2T+ | 432 | 131 | 32 | 826 | 264 | 59 | 52.3 | 49.5 | 54.2 | 2.97 | 1.84 | 1.66 |
| N-Ps2T- | 428 | 136 | 30 | 811 | 276 | 49 | 52.7 | 49.1 | 62.3 | 3.09 | 1.57 | 1.14 |
| N-Ps3T+ | 446 | 183 | 55 | 789 | 330 | 90 | 56.4 | 55.3 | 61.3 | 3.03 | 1.70 | 1.31 |
| N-Ps3T- | 406 | 193 | 61 | 805 | 399 | 86 | 50.4 | 48.3 | 70.3 | 2.81 | 2.01 | 1.54 |
| N-L1T+ | 353 | 120 | 23 | 822 | 198 | 49 | 43.0 | 60.6 | 47.4 | 2.67 | 2.13 | 1.20 |
| N-L1T- | 408 | 230 | 73 | 929 | 423 | 99 | 44.0 | 54.2 | 73.6 | 2.45 | 2.42 | 1.17 |
| N-L2T+ | 433 | 131 | 23 | 827 | 212 | 42 | 52.4 | 62.0 | 54.8 | 2.72 | 1.37 | 1.05 |
| N-L2T- | 401 | 130 | 44 | 753 | 222 | 63 | 53.3 | 58.2 | 70.1 | 2.69 | 1.35 | 1.02 |
| N-L3T+ | 546 | 229 | 70 | 1,133 | 503 | 117 | 48.2 | 45.4 | 59.5 | 3.55 | 2.34 | 1.97 |
| N-L3T- | 313 | 97 | 27 | 753 | 182 | 42 | 41.6 | 53.4 | 63.7 | 1.47 | 0.70 | 0.61 |
| C-S1T+ | 378 | 219 | 71 | 648 | 357 | 94 | 58.3 | 61.3 | 76.3 | 4.90 | 4.77 | 3.75 |
| C-S1T- | 173 | 123 | 30 | 284 | 170 | 43 | 61.0 | 72.5 | 70.2 | 3.59 | 3.69 | 2.93 |
| C-S2T+ | 113 | 57 | 17 | 186 | 86 | 26 | 60.6 | 66.9 | 62.4 | 2.13 | 2.18 | 1.92 |
| C-S2T- | 711 | 384 | 103 | 1,187 | 586 | 154 | 59.9 | 65.5 | 67.0 | 5.29 | 4.85 | 4.31 |
| C-S3T+ | 315 | 223 | 45 | 525 | 354 | 73 | 60.0 | 63.1 | 62.3 | 3.91 | 3.91 | 3.38 |
| C-S3T- | 769 | 593 | 177 | 1,799 | 1,112 | 254 | 42.8 | 53.4 | 69.8 | 6.85 | 6.67 | 4.60 |
| C-Re1T+ | 626 | 408 | 135 | 1,107 | 706 | 189 | 56.5 | 57.7 | 71.3 | 6.64 | 5.65 | 3.92 |
| C-Re1T- | 476 | 357 | 125 | 795 | 506 | 181 | 59.8 | 70.6 | 69.1 | 4.12 | 3.81 | 2.50 |
| C-Re2T+ | 923 | 635 | 207 | 2,014 | 1,210 | 275 | 45.8 | 52.5 | 75.1 | 6.62 | 5.97 | 4.37 |
| C-Re2T- | 891 | 594 | 180 | 1,781 | 1,168 | 272 | 50.0 | 50.9 | 66.2 | 5.97 | 4.90 | 3.81 |
| C-Re3T+ | 1,021 | 621 | 174 | 2,174 | 1,164 | 236 | 47.0 | 53.3 | 73.6 | 5.61 | 4.73 | 3.48 |
| C-Re3T- | 1,505 | 1,154 | 289 | 3,471 | 2,230 | 382 | 43.3 | 51.7 | 75.8 | 7.50 | 6.83 | 4.86 |
| C-Ps1T+ | 174 | 54 | 22 | 288 | 102 | 31 | 60.3 | 53.2 | 69.6 | 3.54 | 2.44 | 2.16 |
| C-Ps1T- | 175 | 50 | 11 | 254 | 87 | 16 | 68.7 | 57.9 | 69.1 | 1.97 | 1.84 | 1.21 |
| C-Ps2T+ | 492 | 125 | 19 | 905 | 197 | 39 | 54.3 | 63.6 | 48.5 | 3.00 | 1.95 | 1.36 |
| C-Ps2T- | 718 | 190 | 32 | 1,249 | 305 | 41 | 57.5 | 62.2 | 77.9 | 3.77 | 1.94 | 1.34 |
| C-Ps3T+ | 454 | 111 | 23 | 746 | 191 | 39 | 60.8 | 58.1 | 57.8 | 3.34 | 1.85 | 1.38 |
| C-Ps3T- | 229 | 123 | 30 | 408 | 190 | 48 | 56.3 | 64.8 | 62.6 | 3.02 | 3.20 | 2.08 |
| C-L1T+ | 455 | 158 | 35 | 829 | 258 | 68 | 54.8 | 61.2 | 51.4 | 4.23 | 3.05 | 2.38 |
| C-L1T- | 464 | 139 | 24 | 1,013 | 245 | 27 | 45.8 | 56.8 | 87.3 | 3.70 | 3.04 | 2.27 |
| C-L2T+ | 250 | 37 | 18 | 384 | 92 | 29 | 65.0 | 39.9 | 62.8 | 1.51 | 0.27 | 0.24 |
| C-L2T- | 325 | 93 | 24 | 634 | 153 | 42 | 51.3 | 60.9 | 56.9 | 3.60 | 2.57 | 2.14 |
| C-L3T+ | 406 | 143 | 31 | 729 | 221 | 53 | 55.7 | 64.8 | 58.5 | 4.12 | 3.32 | 2.43 |
| C-L3T- | 320 | 121 | 29 | 524 | 203 | 40 | 61.1 | 59.5 | 72.6 | 3.12 | 2.60 | 1.09 |

^a^Sample abbreviations indicate: (1) country (N- = Nicaragua, C- = Costa Rica), (2) microenvironment (S = rhizosphere soil, Re = endorhiza, Ps = pseudostem, L = leaves), (3) farm (1, 2, 3), and (4) agroforestry conditions (T+ = with trees, T- = without trees).

^b^quality reads were normalized to the same number of sequences.

^c^averaged rarefaction curves are depicted in Figure S2.

**Table S2** Statistical comparisons of the gammaproteobacterial communities between banana plants grown in Nicaragua and Costa Rica.

|  | **Agroforestry** | **Without Trees** |
| --- | --- | --- |
| **Rhizosphere** | 0.111 | 0.098 |
| **Endorhiza** | 0.205 | 0.687 |
| **Pseudostem** | 0.090 | 0.312 |
| **Leaves** | 0.090 | 0.236 |

p values of individual microenvironments for pairwise comparisons between plants grown in Nicaragua and Costa Rica ascertained by adonis test based on weighted UniFrac distances.

**Table S3** Statistical comparisons of the gammaproteobacterial communities of banana plants grown with and without agroforestry.

|  | **Nicaragua** | **Costa Rica** |
| --- | --- | --- |
| **Rhizosphere** | 0.796 | 0.903 |
| **Endorhiza** | 1.000 | 0.830 |
| **Pseudostem** | 0.396 | 0.280 |
| **Leaves** | 0.387 | 0.501 |

p values of individual microenvironments for pairwise comparisons between plants grown with and without associated trees ascertained by adonis test based on weighted UniFrac distances.


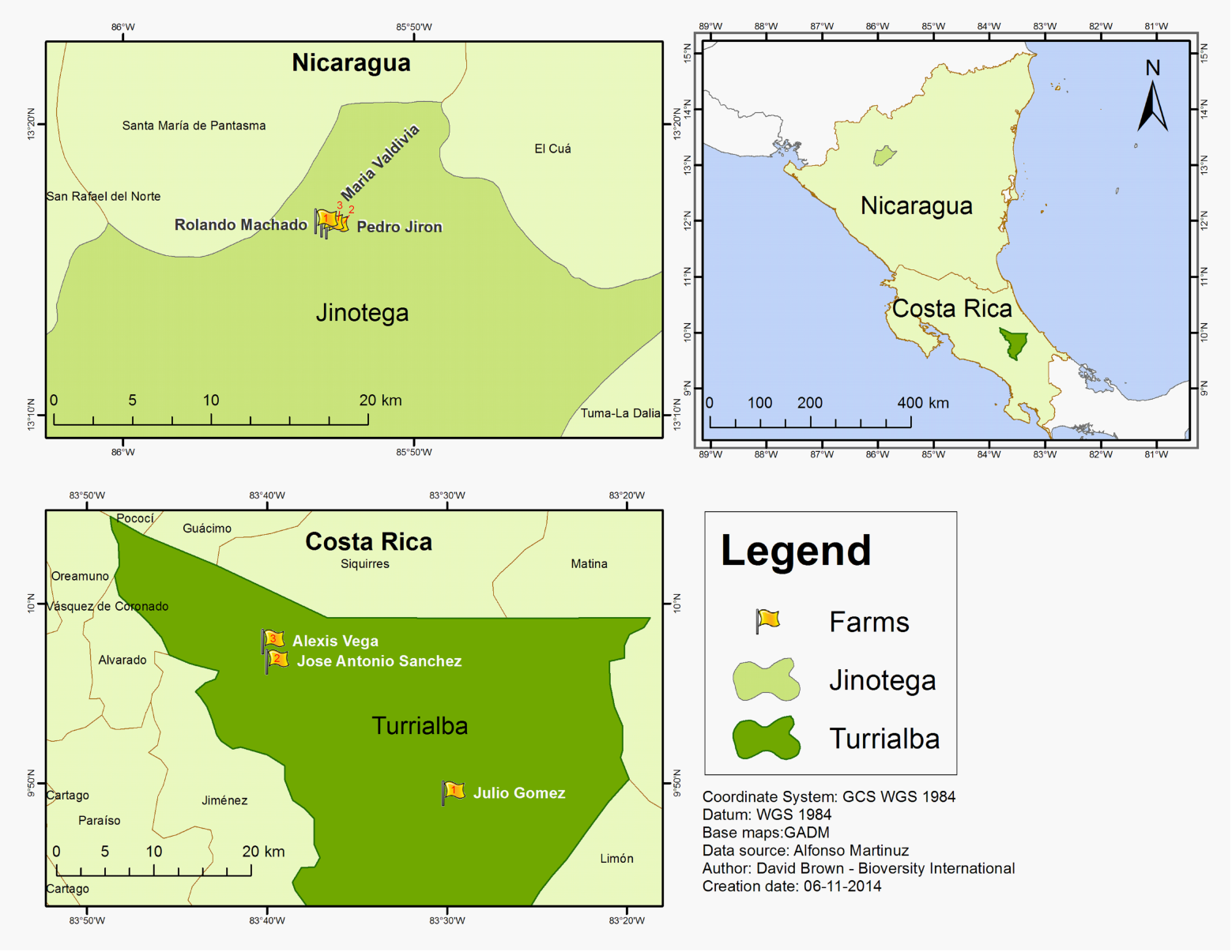


**Figure S1** Locations of the individual farms in Nicaragua and Costa Rica. On each farm, samples were taken from *Musa acuminata* Colla (AAA group) cultivar Gros Michel.


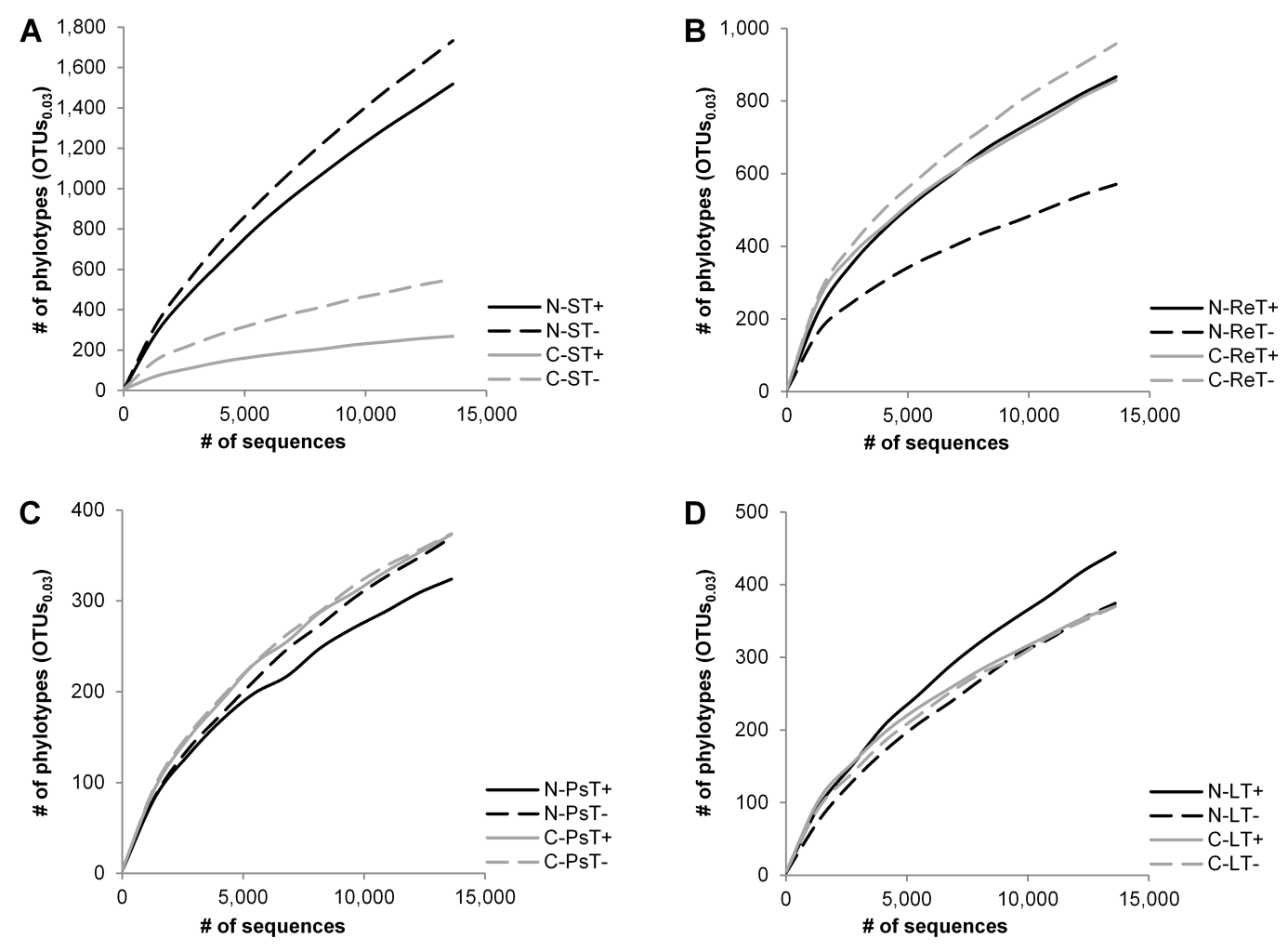


**Figure S2** Rarefaction analysis of averaged *Gammaproteobacteria* specific 16S rRNA gene sequence libraries at a dissimilarity level of 3% divided into rhizosphere soil (A), endorhiza (B), pseudostem (C), and foliage samples (D). N- = Nicaragua, C- = Costa Rica; S = rhizosphere soil, Re = endorhiza, Ps = pseudostem, L = leaves; T+ = with trees, T- = without trees.
